# Palaeopathological Survey of a Population of *Mapusaurus* (Theropoda: Carcharodontosauridae) from the Late Cretaceous Huincul Formation, Argentina

**DOI:** 10.1371/journal.pone.0063409

**Published:** 2013-05-15

**Authors:** Phil R. Bell, Rodolfo A. Coria

**Affiliations:** 1 Pipestone Creek Dinosaur Initiative, County of Grande Prairie No. 1, Clairmont, Alberta, Canada; 2 Universidad Nacional de Río Negro, Sede Alto Valle, Gral Roca, Río Negro, Argentina; Ludwig-Maximilians-Universität München, Germany

## Abstract

Paleoepidemiology (the study of disease and trauma in prehistoric populations) provides insight into the distribution of disease and can have implications for interpreting behavior in extinct organisms. A monospecific bonebed of the giant carcharodontosaurid *Mapusaurus* (minimum number of individuals = 9) from the Cañadón del Gato site, Neuquén Province, Argentina (Cenomanian) provides a rare opportunity to investigate disease within a single population of this important apex predator. Visual inspection of 176 skeletal elements belonging to a minimum of nine individuals yielded a small number of abnormalities on a cervical vertebra, two ribs, pedal phalanx, and an ilium. These are attributed to traumatic (two cases), infectious (two cases) and anomalous (one case) conditions in a minimum of one individual. The emerging picture for large theropod (abelisaurids, allosaurids, carcharodontosaurids, tyrannosaurids) populations suggests that 1) osseous abnormalities were relatively rare (7–19% of individuals) but consistently present, and 2) trauma was a leading factor in the frequency of pathological occurrences, evidence of an active, often perilous lifestyle.

## Introduction

*Mapusaurus* is a large (up to 11 m long) carcharodontosaurid, comparable in size to the largest known theropods including *Giganotosaurus* and *Tyrannosaurus*. Hundreds of disarticulated elements of *Mapusaurus* were collected from the type locality in a single bonebed at the Cañadón del Gato site 20 km southwest of the town of Plaza Huincul, Neuquén Province, Argentina during successive fieldtrips from 1996 to 2000. The material, collected from the Late Cretaceous (Cenomanian) Huincul Formation, is relatively poorly preserved; bone surfaces are frequently weathered and individual elements exhibit differential compaction. Nevertheless, this monospecific assemblage has important implications regarding the ecology and social behavior of these animals [1], [2].

Remains from the Cañadón del Gato bonebed suggest the presence of a minimum of seven-to-nine individuals ranging in length from 5.5 m to 11 m. All elements were found disarticulated and were subject to a complex taphonomic history of decomposition, trampling, reworking, and final burial [1]. The depositional environment has been interpreted as an ephemeral and/or seasonal channel deposit within a semiarid or arid palaeoenvironment [3].

Close visual inspection of prepared elements from the Cañadón del Gato bonebed identify multiple pathological elements not mentioned in the original osteological description of *Mapusaurus* [1]. The presence of osteological abnormalities from this site provides a rare opportunity to examine the types and frequency of pathological changes in a single theropod population. Palaeoepidemiological reports have been limited to three such studies on theropods: the abelisaurid *Majungasaurus crenatissimus* from Madagascar [4]; the tyrannosaurid *Albertosaurus sarcophagus* from Dry Island in Alberta, Canada [5]; and perhaps most spectacularly in *Allosaurus fragilis* from the Cleveland-Lloyd quarry, Utah [6]. Such sites offer insights into the susceptibility of certain taxa to disease and relative frequencies of injury that have implications for behavior and survival of these animals [4].

With the exception of bite marks [7], pathological conditions have not been noted previously in carcharodontosaurids. The purpose of this paper is to document the types and frequencies of pathological changes in *Mapusaurus* from the Cañadón del Gato bonebed, which are compared to the slowly-growing list of palaeoepidemiological studies for large theropods from across the globe.

## Materials and Methods

A total of 176 catalogued cranial and postcranial elements from the *Mapusaurus* bonebed at Cañadón del Gato (Museo Carmen Funes, Paleontología de Vertebrados [Plaza Huincul, Neuquén, Argentina], MCF-PVPH-108 series) were visually inspected for osteological abnormalities. All material is accessioned in the vertebrate palaeontology collection at Museo Carmen Funes, Plaza Huincul (Neuquén, Argentina). Elements were compared with a subset of ‘normal’ *Mapusaurus* bones from the same locality to assess for potential pathological changes. A minimum of seven individuals was determined from metatarsals (total body lengths ranging from 6 to 7.3 m) [1] although other skeletal material reveals the presence of at least two additional individuals (5.5 and 9.8 m in length, respectively) bringing the total minimum number of individuals from the bonebed up to nine [1]. It should be noted that most of the bones from the *Mapusaurus* bonebed are poorly preserved. Bone surfaces are generally highly fractured and elements are often incomplete, potentially obscuring other bone abnormalities during our observations.

Following each description, an etiological hypothesis and differential diagnoses are offered for each pathological element based on comparisons available from human and modern vertebrate pathology literature. Although this approach does not follow the desired extant phylogenetic bracket [8] it is applicable because: 1. Directly comparable osteological material (e.g. avian or crocodilian examples) is either poorly known or unavailable, and 2. It provides a testable hypothesis for future examinations. Because of these limitations, however, we use a modified version of Hanna’s [6] classification of bone abnormalities based on broader etiology: traumatic (resulting from injury), infectious (resulting from infection = osteomyelitis), traumatic-infectious (injury followed by secondary infection), developmental (resulting from growth disturbance during development), and anomalous (of uncertain origin). These categories are useful because they avoid the temptation to over interpret the evidence. While bone disorders in non-avian theropods may closely resemble conditions in living vertebrates, such as human patients (uniformitarianism remains a key assumption in palaeopathology), it is still unclear precisely how dinosaur bone reacted to disease [6] and caution is recommended when attempting to interpret evidence in dinosaur palaeopathology. No permits were required for the described study, which complied with all relevant regulations.

## Results

### Cervical Vertebra

**Figure 1:**
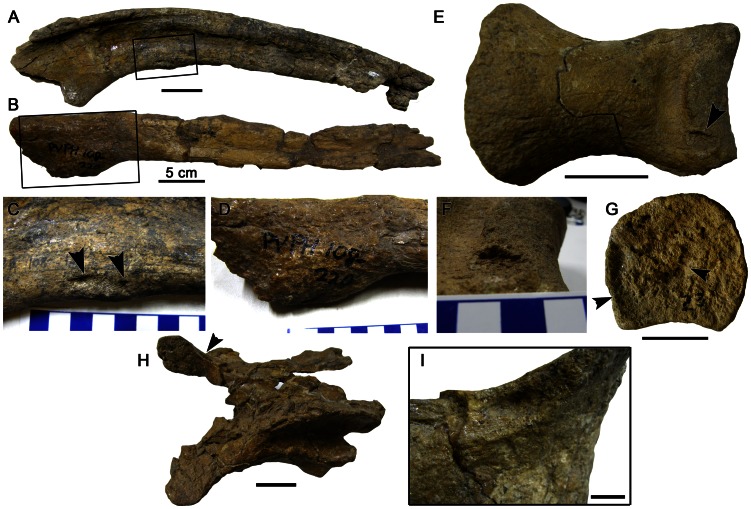
Pathological postcranial elements in *Mapusaurus roseae*. A. Right dorsal rib (MCF-PVPH 108–175); B. Dorsal rib (MCF-PVPH 108–220); C. Close up of boxed region in A showing erosions (arrows) on overtubulated area; D. Close up of boxed region in B; E. ?Left pedal phalanx III-1 (MCF-PVPH 108–23) showing marginal erosion (arrow) on distal articular surface; F. Oblique view of lesion identified in E; G. Proximal articular view of MCF-PVPH 108–23 showing elongate articular surface irregularities (arrows); H. Mid-caudal neural arch (MCF-PVPH 108–90) in dorsal view showing location of erosion (arrow) on right prezygapophysis; I. Posterolateral view of lesion identified in H. Scales in A, B, E, G, H  = 5 cm; scale in I  = 1 cm. Scale increments in C, D, F  = 1 cm.

An unfused cervical neural arch (MCF-PVPH 108–90) preserves a single pathological cavity on the posterolateral margin of the right prezygapophyseal facet (Fig. 1H,I). The lesion is tear-drop shaped (20×9 mm), tapering posteriorly, and up to 6 mm deep. Perilesional growth is absent and the edges are rounded. The erosion is localized and does not encroach onto the articular surface of the prezygapophysis. Rheumatoid arthritis presents as periarticular erosions with smooth inner walls and rounded edges similar to that in MCF-PVPH 108–90. New bone growth is notably absent. However, rheumatoid arthritis does not affect the zygapophyses in human subjects [9]. Infection can cause osseous erosions; however, this is usually accompanied by exuberant bone growth, which is absent in MCF-PVPH 108–90. Some forms of bacterial infection, such as tuberculosis, produce erosions with minimal new bone formation [9], [10] and, unlike rheumatoid arthritis, can affect the zygapophyses. Because tuberculosis and other infections are named for a specific bacterium (e.g. *Mycobacterium tuberculosis*), we refrain from giving MCF-PVPH 108–90 a more specific diagnosis and simplify classify the lesion as infectious.

### Dorsal Ribs

MCF-PVPH 108–175 is an incomplete right dorsal rib that lacks both the head and the distal half of the element. The rib shaft is weakly expanded (overtubulated) along its medial edge approximately 10 cm from the proximal end of the bone (Fig. 1A). The expanded region is approximately 3 cm long and grades imperceptibly into the surrounding unaffected bone. The surface of the affected area also preserves an irregular-walled (i.e. not smooth), oblong lesion that does not appear to have exposed the deeper trabecular bone. The lesion measures approximately 27 mm long and 9 mm wide (Fig. 1C). Slight overtubulation of the rib is indicative of a well-healed fracture (trauma) and the presence of an erosive lesion may be indicative of secondary suppurative (pus forming) osteomyelitis.

A second incomplete dorsal rib of uncertain position (MCF-PVPH 108–220) exhibits substantial overtubulation of the shaft (Fig. 1B,D). The rib is lacking both proximal and distal ends and appears to have broken (postmortem) part way through the pathologic region. The affected area is an elongate bulge and, as preserved, measures more than 7 cm in maximal length (Fig. 1D). This bulge is restricted to one edge (?medial) of the element and grades smoothly into the surrounding unaffected bone. The bone surface along the expanded area does not differ significantly from the surrounding unaffected parts of the element; however, there is a faint interfingering of bony spicules that meander across the surface of the lesion roughly perpendicular to the long axis of the element. The smooth texture, overtubulation, and interfingering of bone is characteristic of an incomplete but well-healed fracture.

### Ilium

**Figure 2:**
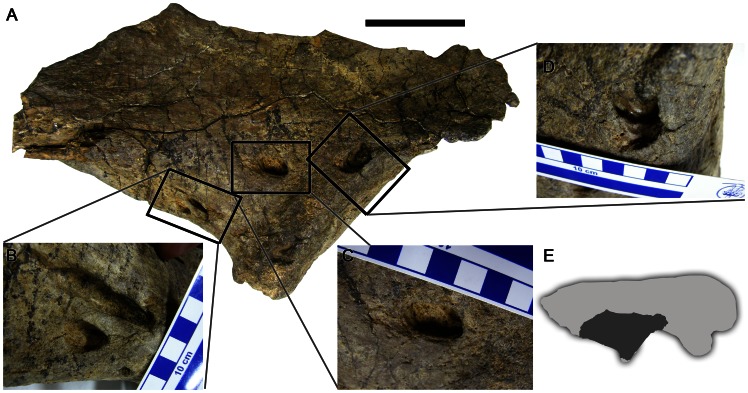
Abnormalities in a partial *Mapusaurus roseae* right ilium (MCF-PVPH 108–181). A. Lateral view of acetabular region; B–D, close ups of elliptical lesions; E, *Mapusaurus* right ilium showing position (dark grey) of MCF-PVPH 108–181. Scale in A  = 10 cm. Scale increments in close-up photographs  = 1 cm.

MCF-PVPH 108–181 is a partial right ilium comprising the ventral part of the body including the anterior part of the acetabulum, ischiadic peduncle, and the proximal segment of the postacetabular process (Fig. 2). Five erosional lesions are preserved on the ventrolateral surface of the ilium in the vicinity of the acetabulum and brevis shelf. All are deep (up to and exceeding 10 mm in depth), elliptical in outline, and penetrate obliquely into the bone. Because lesions penetrate at an angle to the bone surface, the margin is often marked by a sharp ‘lip’. Lesions are of variable size ranging from 8 mm to 50 mm in maximum dimension. Perilesional bone growth is absent. The internal surfaces of the cavities are smooth and do not reveal trabecular bone (Fig. 2B–D).

The ilium of *Mapusaurus* is highly pneumatic^1^ and it is possible that one or two of these fenestrae are in fact continuous with the pneumatic diverticulae. Coria and Currie [1] also noted the presence of the pits within the margins of the brevis fossa in MCF-PVPH 108–181 and a second specimen from the bonebed (MCF-PVPH 108–245) suggesting they may be connected to the lateral caudofemoralis musculature. However, the location of the other lesions in MCF-PVPH 108–181 suggest they are pathological in origin.

Smooth-walled erosions similar to those in MCF-PVPH 108–181 may form as a result of osteomyelitis, fungal disease, bone tumors, or cancer. Pus-draining sinuses that form as a result of infection (osteomyelitis) are typically accompanied by periosteal reaction and rapidly-formed new bone. The latter is characterized by disorganized bone texture. Fungal diseases (such as coccidioidomycosis and blastomycosis) can also produce erosions; however, these are accompanied by short, blunt spicules of new bone (osteophytes) and periosteal reaction [10]. The absence of reactive bone and periosteal reaction in MCF-PVPH 108–181 argues against either fungal disease or osteomyelitis as a possible diagnosis. Several types of malignant tumors (cancer) produce lesions similar to those seen in MCF-PVPH 108–181. Lesions associated with eosinophilic granuloma are predominantly localized in human subjects to the vertebrae and pelvis where they occur as ‘space occupying masses’ with little reactive bone growth [11]. The lesions have effaced (indistinct) trabeculae and periosteal reaction is variably absent. Myeloma produces sharply-defined, spheroid erosions with smooth borders and effaced trabeculae [12]. Lesions are variable in size and secondary bone formation and periosteal reaction does not occur. When cancer spreads to a secondary location (metastatic cancer), it often produces multiple holes of variable size [11]. These lesions are elliptical with loss of trabecular bone and periosteal reaction is usually absent [12]. Metastatic cancer and myeloma both produce lesions that coalesce. Total absence of remodeling in myeloma distinguishes it from metastatic cancer [12]. Hematologic cancer (leukemia) produces large numbers of small (up to several millimetres in diameter), smooth-walled lesions [13]. The similarity of perforative lesions in MCF-PVPH 108–181 to tumor-related neoplasia suggests a possible etiology for such abnormalities in *Mapusaurus*; however, bone cysts, parasitic infection, or some other unidentified disease process cannot be ruled out. Therefore the exact etiology is unknown and the lesions are diagnosed as anomalous.

### Pedal Phalanx

A single pedal phalanx III-1 (MCF-PVPH 108–23) bears a number of erosive lesions on the proximal articular surface and the dorsal margin of the distal articular surface (Fig. 1E–G). Because of the symmetry of this element, it was not possible to identify whether MCF-PVPH 108–23 is from the left or the right pes. The entire surface of the proximal articular surface is uneven and pockmarked with erosive lesions giving it an overall disorganized appearance. Disruption of the bone surface is limited to the articular surface and reactive bone growth is altogether absent. The largest erosive features are several centimeters long with irregular walls up to 5 mm deep. The interior of the deepest cavities reveals trabecular bone with a unique ‘stringy’ texture owing to the parallel orientation of the trabeculae (Fig. 1G). A circular erosion also occurs on the dorsal margin of the distal articular surface (Fig. 1F). Some marginal thickening (sclerosis) of the periosteum is present.

Articular surface irregularities may be cause by a variety of disorders including osteochondrosis, osteoarthritis, rheumatoid arthritis, avascular necrosis ( = osteochondritis desiccans, osteochondrosis desiccans, osteonecrosis), and also infection. In osteochondrosis, the resulting lesions are smooth-walled with sharply-delimited margins [14] contrasting with the overall disorganized texture of the articular surface in MCF-PVPH 108–23. Osteoarthritis is linked to disruption of the cartilaginous lining of the joint and is non-erosive. Osteophyte formation around the periphery of the joint, characteristic of osteoarthritis, is absent in MCF-PVPH 108–23. Rheumatoid arthritis (an erosive form of arthritis) also produces similar erosions to those in MCF-PVPH 108–23. Erosions are polyarticular in distribution and occur at the margin of the joint capsule. Compromised blood supply to the bone leads to the death and subsequent loss of bony tissue in avascular necrosis [15], [16]. Structural weakening leads to the collapse of the articular surface (focal subsidence) resulting in broad depressions and an overall uneven bone surface areas that lacks restorative bone growth similar to the erosions in MCF-PVPH 108–23. Joint infection can cause the loss of bone as a result of inflammation and decreased blood flow, which is accompanied by rapid formation of new bone that has a fibrous, disorganized texture. Pyogenic (pus-generating) infection also results in the formation of pus-draining sinus tracts (cloacae). Exuberant reactive bone growth and cloacae are absent in MCF-PVPH 108–23. Conversely, non-pyogenic forms of bacterial infection (e.g. tuberculosis), can result in marginal erosions with minimal or no reactive bone formation. Lesions form at the margin of the join capsule and invade the underlying subchondral bone. Minor reactive bone is present around the periphery of lesions [10], similar to MCF-PVPH 108–23. Lesions in MCF-PVPH 108–23 are therefore diagnosed as infectious.

## Discussion

Pathological elements from the *Mapusaurus* bonebed are classified as traumatic (dorsal ribs MCF-PVPH 108–220, MCF-PVPH 108–175), infectious (cervical neural arch MCF-PVPH 108–90; pedal phalanx MCF-PVPH 108–23) and anomalous (ilium MCF-PVPH 108–181). Healed fractures are among the most commonly reported abnormalities in theropods typically localized on ribs and long bones [17]. The identification of two broken and rehealed ribs in the *Mapusaurus* sample from Cañadón del Gato is not surprising and in both cases represent ‘old’ injuries from which the animal(s) clearly recovered. In another palaeoepidemiologic study, rehealed fractures (affecting ribs and gastralia) were among the most common pathologies in a population of *Albertosaurus* from Alberta [5]. Rehealed fractures were also common in *Allosaurus* from the Cleveland-Lloyd quarry [6] but were not confirmed from the *Majungasaurus* bonebed reported by Farke and O’Connor [4]. Such activity-related injuries are directly related to behavior and have been cited as evidence of predaceous or at least active lifestyles in theropods [17] and there seems little doubt that *Mapusaurus* led an active life. The absence of healed fractures in *Majungasaurus* is likely a collection bias rather than of actual biological significance. The infectious lesions on the cervical neural arch and pedal phalanx were unlikely to have been debilitating at least at the time of death; however, had the animal(s) lived, prolongation to bacterial infection may have spread to other joints and severely hampered mobility (especially with respect to the pedal phalanx), with potentially fatal results. Conversely, the extensive erosions on the ilium were probably more painful but not responsible for the death of the animal (as indicated by the taphonomic setting [1], [3]).

Visual inspection of 176 skeletal elements (MNI = 9) of the carcharodontosaurid *Mapusaurus rosea* from the Cañadón del Gato bonebed yielded a small number (n = 5; 2.8% of elements) of pathological bones belonging to at least one individual (11.1% of individuals). These constitute the first reported pathological conditions in any carcharodontosaurid. The frequency of pathological elements in *Mapusaurus* are in agreement with previous studies on theropod populations (Table 1), which indicate relatively low numbers of skeletal abnormalities in a given population. Although the number of palaeoepidemiological studies is limited, these studies form an important database for assessing the frequency and types of skeletal abnormalities in theropod populations. These preliminary results provide an estimate for ‘normal’ incidents of disease and injury of between 7 and 19 per cent in a given population. These numbers will no doubt be refined with additional population surveys. Indeed, numerous theropod bonebeds are found worldwide and require palaeopathological investigation that will contribute significantly to the understanding of palaeoepidemiology within this clade (Table 2).

**Table 1:** Palaeoepidemiology of Large Theropods.

Taxon	No. of elementsexamined	TotalMNI	No. of pathologicalelements (%)	PathologicalMNI	Pathological %of population	Reference	Trauma	infectious	Traumatic-infectious	Developmental	Anomalous
*Allosaurus fragilis*	?	40+	30 (?)	?	?	Hanna [6]	7	7	2	1	1
*Majungasaurus crenatissimus*	181	21	8 (4.4)	4	19	Farke andO’Connor [4]	1*		1*		2
*Albertosaurus sarcophagus*	190	26	6 (4.7)	2	7.7	Bell [5]	4				3
*Mapusaurus rosea*	176	9	5 (2.8)	1	11.1	Bell and Coria(this study)	2	1			2

* Traumatic and traumatic-infectious conditions were tentatively identified by Farke and O’Connor [4] although other causes could not be positively ruled out.

**Table 2:** Theropod Bonebeds as Potential Candidates for Palaeoepidemiological Studies.

Taxon	Site	Age	MNI	References
*Archaeornithomimus asiaticus*	Iren Dabasu, China	Late Cretaceous	10+	Currie and Eberth [2]
*Avimimus* sp.	Nemegt, Mongolia	Late Cretaceous	10	Currie et al. [18]
*Coelophysis bauri*	Ghost Ranch, USA	Triassic	1000+	Colbert [19]
Coelophysoid new taxon	Arizona, USA	Early Jurassic	16	Tykoski [20]
*Daspletosaurus* sp.	Montana, USA	Late Cretaceous	3	Currie et al. [21]
*Deinonychus antirrhopus*	YPM 64–75, Montana, USA	Early Cretaceous	4	Ostrom [22]
*Deinonychus antirrhopus*	MOR CL-103, Montana, USA	Early Cretaceous	6 to 8	Listed in Currie and Eberth [2]
*Falcarius utahensis*	Crystal Geyser Quarry, USA	Early Cretaceous	10+	Kirkland et al. [23]
*Fukuiraptor kitadaniensis*	Fukui Prefecture, Japan	Early Cretaceous	14	Currie and Azuma [24]
*Masiakasaurus knopfleri*	Majunga, Madagascar	Late Cretaceous	6	Sampson et al. [25]
*Ornithomimus edmontonicus*	Dry Island, Alberta, Canada	Late Cretaceous	3	Sternberg fieldnotes 1926
*Saurophaganax maximus*	Kenton, Oklahoma, USA	Late Jurassic	4	Chure [26]
*Sinornithomimus dongi*	Near Ulan Suhai, China	Late Cretaceous	27	Kobayashi and Lu [27]
*Syntarsus kayentakatae*	Arizona, USA	Early Jurassic	4	Rowe [28]
*Syntarsus rhodesiensis*	Chitake River, Zimbabwe	Triassic	26	Raath [29]
*Tarbosaurus baatar*	Hermiin Tsav, Mongolia	Late Cretaceous	3	P.J. Currie fieldnotes
*Troodon formosus*	Jack’s Birthday Site, Montana, USA	Late Cretaceous	4	Varricchio [30]
*Tyrannosaurus rex*	Buffalo, South Dakota, USA	Late Cretaceous	4	Larson [31]
